# Behavioral Modeling Based on Probabilistic Finite Automata: An Empirical Study †

**DOI:** 10.3390/s16070958

**Published:** 2016-06-24

**Authors:** Cristina Tîrnăucă, José L. Montaña, Santiago Ontañón, Avelino J. González, Luis M. Pardo

**Affiliations:** 1 Departamento de Matemáticas, Estadística y Computación, Universidad de Cantabria, Santander 39005, Spain; joseluis.montana@unican.es (J.L.M.); luis.m.pardo@gmail.com (L.M.P.); 2 Department of Computer Science, Drexel University, Philadelphia, PA 19104, USA; santi@cs.drexel.edu; 3 Department of Computer Science, University of Central Florida, Orlando, FL 32816, USA; gonzalez@ucf.edu

**Keywords:** learning from observation, behavioral recognition, behavioral cloning, probabilistic finite automaton, ambient intelligence, virtual agents

## Abstract

Imagine an agent that performs tasks according to different strategies. The goal of Behavioral Recognition (BR) is to identify which of the available strategies is the one being used by the agent, by simply observing the agent’s actions and the environmental conditions during a certain period of time. The goal of Behavioral Cloning (BC) is more ambitious. In this last case, the learner must be able to build a model of the behavior of the agent. In both settings, the only assumption is that the learner has access to a training set that contains instances of observed behavioral traces for each available strategy. This paper studies a machine learning approach based on Probabilistic Finite Automata (PFAs), capable of achieving both the recognition and cloning tasks. We evaluate the performance of PFAs in the context of a simulated learning environment (in this case, a virtual Roomba vacuum cleaner robot), and compare it with a collection of other machine learning approaches.

## 1. Introduction

Modern training, entertainment and education applications make extensive use of autonomously controlled virtual agents or physical robots. In these applications, the agents must display complex intelligent behaviors that, until recently, were only shown by humans. Driving simulations, for example, require having vehicles moving in a realistic way in the simulation, while interacting with other virtual agents as well as humans. Likewise, computer games require artificial characters or opponents that display complex intelligent behaviors to enhance the entertainment factor of the games. Manually creating those complex behaviors is usually expensive. For example, the artificial intelligence for the two virtual characters in the game *Façade* [1] took more than five person-years to develop. Additionally, the required knowledge to create these behaviors is often tacit. In other words, humans that are proficient in the task at hand have difficulties articulating this knowledge in an effective manner so that it can be included into the agent’s behavior. For example, when asked how hard to apply the brakes in a car when approaching a traffic light, most human practitioners would be unable to find appropriate words to describe the experience. On the other hand, showing how to do it is much easier.

Another example comes from the United States Department of Defense, which was one of the first official institutions to recognize the value of intelligent virtual agents that could reliably act as intelligent opponents, friendly forces and neutral bystanders in training simulations. The availability of such agents permitted them to avoid using human experts, a scarce and expensive resource, who had been used to manually control such entities in past training sessions. Semi-Automated Forces (SAF) were the first attempt at developing such software agents, where simulated enemy and friendly entities inhabit the virtual training environment and act to combat or support war-fighters in training sessions. Recent advances in the closely related area of Computer-Generated Forces (CGF) indicate important progress in simulating behaviors that are more complex, but at the cost of operational efficiency. However, CGF models are still very difficult to build, validate and maintain.

An attractive and promising alternative to handcrafting behaviors is to automatically generate them through machine learning techniques. The problem of automatically generating behaviors has been studied in artificial intelligence at least from two different perspectives. The first one is Reinforcement Learning (RL), which focuses on learning from experimentation. The second one is Learning from Observation (LfO), focusing on learning by observing sample traces of the behavior to be learned. Reinforcement Learning is by far the better known and studied of the two, but presents several open problems like scalability and generalization. Furthermore, RL does not lend itself to learn human-like behaviors, given its inherent nature toward optimization. We believe that LfO offers a promising and computationally tractable approach to achieve human-like behaviors with high fidelity, at an affordable cost, and with a reasonable level of generalization. Although LfO has already shown some success in learning behaviors requiring tacit knowledge, many open problems remain in the field.

Some of the early work on the field refers to a technique called programming by demonstration. For example, Bauer [2] showed how to make use of knowledge about variables, inputs, instructions and procedures in order to learn programs, which basically amounts to learning strategies to perform abstract computations by demonstration. Programing by demonstration has also been especially popular in robotics [3]. Another early mention of LfO comes from Michalski et al. [4], who define it merely as unsupervised learning. Gonzalez et al. [5] discussed LfO at length, but provided no formalization nor suggested an approach to realize it algorithmically. More extensive work on the more general LfO subject came nearly simultaneously but independently from Sammut et al. [6] and Sidani [7]. Fernlund et al. [8] used LfO to build agents capable of driving a simulated automobile in a city environment. Pomerleau [9] developed the Autonomous Land Vehicle in a Neural Network (ALVINN) system that trained neural networks from observations of a road-following automobile in the real world. Moriarty and Gonzalez [10] used neural networks to carry out LfO for computer games. Könik and Laird [11] introduced LfO in complex domains with the State, Operator and Result (SOAR) system, by using inductive logic programming techniques. Other significant work done under the label of learning from demonstration has emerged recently in the Case-Based Reasoning community. For example, Floyd et al. [12] present an approach to learn how to play RoboSoccer by observing the play of other teams. Ontañón et al. [13] use learning from demonstration for real-time strategy games in the context of Case-Based Planning. Another related area is that of Inverse Reinforcement Learning [14], where the focus is on reconstructing the reward function given optimal behavior (i.e., given a policy, or a set of trajectories). One of the main problems here is that different reward functions may correspond to the observed behavior, and heuristics need to be devised to consider only those families of reward functions that are interesting. In this paper, we present an approach to LfO based on Probabilistic Finite Automata (PFAs). PFAs are interesting because, in addition to learning a model of the desired behavior, they can also assess the probability that a certain behavior was generated by a given PFA. For that reason, given a training set consisting of traces with observations that come from an agent exhibiting different behaviors, PFAs can be used for two different tasks. The first one is behavioral recognition: a PFA can be trained from the traces of each one of these behaviors (one PFA per different behavior). Then, given a new, unseen, behavioral trace from another agent, these PFAs can be used to assess the probability that this new behavior has been produced by each one of them. Assuming that each PFA is a good model of each of the behaviors of interest, this effectively corresponds to identifying which of the initial set of behaviors was exhibited by the new agent. The second one is behavioral cloning: by training a PFA with the traces corresponding to the desired behavior, such a PFA can be used to recreate this behavior in a new, unseen, situation. The notion of BC was first introduced in [15] to refer to a form of imitation learning whose motivation is to build a model of the behavior of a human.

The perspective provided by our work is general enough to deal with significant applications such as masquerade detection in computer intrusion, analysis of the task performed by the user in some e-learning activity, classification and prediction of the user behavior in a web user interaction process and, more generally, Activity Recognition (AR). The aim of AR is to recognize the actions and tasks of one or several agents taking as input a sequence of observations of their actions and the state of the environment. Most research in AR concentrates on the recognition of human activities. One goal of Human Activity Recognition (HAR) is to provide information on a user’s behavior that allows computing systems to proactively assist users with their tasks (see [16] for a detailed overview on the subject). Hidden Markov Models are widely used tools for prediction in the context of HAR. Successful applications of these machines include important applications like speech recognition [17] and DNA sequence alignment [18].

The remainder of this paper is organized as follows. In Section 2, we introduce our proposed LfO framework, we explain how this framework can be used for BC and BR, and we propose a set of evaluation metrics to assess the performance of the approach. Then, in Section 3, we report on the experiments conducted in the context of a simulated learning environment (a virtual Roomba vacuum cleaner robot). Finally, concluding remarks and future work ideas are presented in Section 4.

## 2. Methodology

The key idea in Learning from Observation is that there is a learner that observes one or several agents performing a task in a given environment, and records the agent’s behavior in the form of traces. Then, those traces are used by the learner to generalize the observed behavior and replicate it in other similar situations. Most LfO work assumes that the learner does not have access to a description of the task during learning, and thus, the features of the task and the way it is achieved must be learned purely through unobtrusive observation of the behavior of the agent.

Let *B* be the behavior (by, behavior, we mean the control mechanism, policy, or algorithm that an agent or a learner uses to determine which actions to execute over time) of an agent *A*. Our formalization is founded on the principle that behavior can be modeled as a stochastic process, and its elements as random variables dependent on time. Our model includes the following variables (we use the following convention: if *X* is a variable, then we use a calligraphic X to denote the set of values it can take, and lower case x∈X to denote specific values it takes.): the state X∈X of the environment, the unobservable internal state C∈C of the agent and the perceptible action Y∈Y that the agent executes. We interpret the agent’s behavior as a discrete-time process $Z=\{Z_{1},...,Z_{k},...\}$ (which can be either deterministic or stochastic), with state space I=X×C×Y. $Z_{t}=\left(X_{t},C_{t},Y_{t}\right)$ is a multidimensional variable that captures the state of the agent at time *t*, i.e, a description of the environment, the internal state and the action performed at time *t*.

The observed behavior of an agent in a particular execution defines a *trace* *T* of observations: $T=[\left(x_{1},y_{1}\right),\ldots ,\left(x_{m},y_{m}\right)]$, where $x_{t}$ and $y_{t}$ represent the specific perception of the environment and action of the agent at time *t*. The pair of variables $X_{t}$ and $Y_{t}$ represents the *observation* of the agent *A*. We assume that the random variables $X_{t}$ and $Y_{t}$ are multidimensional discrete variables. Under this statistical model, we distinguish three types of behaviors [19]: Type 1 (that includes strict imitation behavior) corresponding to a process that only depends on time (independent of previous states and actions); Type 2 (reactive behavior), where $Y_{t}$ may only depend on the time *t*, the present state $X_{t}$ and the non-observable internal state $C_{t}$; and Type 3 (planned behavior) for the case in which the action $Y_{t}$ depends on the time *t*, on the non-observable internal state $C_{t}$, and on any of the previous states $X_{1},\ldots ,X_{t}$ and actions $Y_{1},\ldots ,Y_{t-1}$.

When a behavior does not explicitly depend on time, we say that it is a *stationary* behavior. In addition, we distinguish between *deterministic* and *stochastic* behavior.

### 2.1. LfO Models

In this article, we model only stationary behaviors of Types 2 and 3 that do not explicitly depend on the internal state. Moreover, we limit the “window” of knowledge in the case of planned behavior to one previous observation (our methodology could be easily extended to allow a larger memory, with the obvious drawback of an increased number of features). This gives rise to three possibilities for the current action $Y_{t}$:

- $Y_{t}$ depends only on current state $X_{t}$ (Model 1);
- $Y_{t}$ depends on previous and current state: $X_{t},X_{t-1}$ (Model 2);
- $Y_{t}$ depends on previous and current state: $X_{t},X_{t-1}$, and on previous action $Y_{t-1}$ (Model 3).

An example of the kind of information available for each of the three models is presented in Table 1, where each row represents a training example, and columns represent features. The last column (*Class*) is the action to be performed, which is what our models try to predict.

Note that some of the actual strategies employed by the agents in our experiments exhibit more complex dependencies (see Section 3.2), but the model of the behavior learned by our approach is restricted to one of the three models presented above.

### 2.2. Machine Learning Tools

We describe in this section the kind of learning machines that we propose for modeling reactive and planned behaviors. Note that the only information we have is a trace with pairs (state, action): we do not know if the trace was produced by a deterministic or a stochastic agent, or whether it uses an internal state. However, we would like to have a mechanism that predicts, in each state, the action to perform.

If the learned strategy is a deterministic one, this can be done via a classifier, using more or less features depending on the model (see Table 1). We experimented with a decision tree (DT) algorithm [20], a probabilistic neural network (PNN) [21], the *k* Nearest Neighbour (kNN) algorithm, the RProp algorithm for multilayer feedforward networks [22] and the Naive Bayes (NB) algorithm. On the other hand, for training stochastic models, we propose the use of PFAs, which we describe below.

PFAs were introduced in the 1960s by Rabin (see [23]) and are still used in several fields of science and technology for modeling stochastic processes in applications such as DNA sequencing analysis, image and speech recognition, human activity recognition and environmental problems, among others. The reader is referred to the work of Dupont et al. [24] for an overview of the basic properties of PFAs and a presentation of their relation with other Markovian models.

Formally, a PFA is a 5-tuple A=(Σ,Q,ϕ,ι,γ), where Σ is a finite alphabet (that is, a discrete set of symbols), *Q* is a finite collection of states, ϕ:Q×Σ×Q⟶[0,1] is a function defining the transition probability (i.e., $\phi (q,a,q^{\prime })$ is the probability of emission of symbol *a* while transitioning to state $q^{\prime }$ from state *q*), ι:Q⟶[0,1] is the initial state probability function and γ:Q⟶[0,1] is the final state probability function. In addition, the following functions, defined over words $\alpha =\left(a_{1},\ldots ,a_{m}\right)\in \Sigma^{*}$ and state paths $\pi =(q_{1},\ldots ,q_{m})\in Q^{*}$, must be probability distributions (Equation (1) when using final probabilities and Equation (2) otherwise):

(1) $$P_{A}\left(\alpha ,\pi \right)=\iota \left(q_{1}\right)\left(\prod_{i=1}^{m-1}\phi \left(q_{i},a_{i},q_{i+1}\right)\right)\gamma \left(q_{m}\right)$$

(2) $${\hat{P}}_{A}\left(\alpha ,\pi \right)=\iota \left(q_{1}\right)\prod_{i=1}^{m-1}\phi \left(q_{i},a_{i},q_{i+1}\right)$$

This implies in particular that the two following functions are probability distributions over $\Sigma^{*}$:

(3) $$P_{A}\left(\alpha \right)=\sum_{q,q^{\prime }}\iota \left(q\right)\phi \left(q,\alpha ,q^{\prime }\right)\gamma \left(q^{\prime }\right)$$

(4) $${\hat{P}}_{A}\left(\alpha \right)=\sum_{q,q^{\prime }}\iota \left(q\right)\phi \left(q,\alpha ,q^{\prime }\right)$$

Note that $P_{A}\left(\alpha \right)$ is the probability of generating word *α* and ${\hat{P}}_{A}\left(\alpha \right)$ is the probability of generating a word with prefix *α*. Here, $\phi (q,\alpha ,q^{\prime })$ is the extension of function ϕ to words with the obvious meaning: the probability of reaching state $q^{\prime }$ from state *q* while generating word *α* (the reader is referred to the work of Dupont et al. [24] for a detailed explanation of Equations (3) and (4)). In many real situations, we are interested in PFAs with no final probabilities, and in this case we use Equation (4).

### 2.3. Training a Probabilistic Finite Automaton

We propose to train a PFA A=(Σ,Q,ϕ,ι,γ) to model an unknown behavior by observing its trace *T*. To this end, we define the alphabet Σ of automaton A to be the set of all actions Y that the agent can perform. The state space *Q* depends on the model: it is either X (Model 1), or X×X (Model 2) or X×Y×X (Model 3).

Training the automaton A from a trace $\left[\left(x_{1},y_{1}\right),\left(x_{2},y_{2}\right),\ldots ,\left(x_{m},y_{m}\right)\right]$ consists of determining the transition probability function values $\left\{\phi \left(q,a,q^{\prime }\right)|q,q^{\prime }\in Q,a\in \Sigma \right\}$ and the initial probability values {ι(q)∣q∈Q} (we opted for a model with no final probabilities). For any state q∈Q, let ♯q be the number of occurrences of symbol *q* in trace *T*. In the case of Model 1, $♯q=|\{i\in \bar{1,m}|x_{i}=q\}|$, for Model 2, if $q=(x,x^{\prime })$, then $♯q=|\{i\in \bar{2,m}|x_{i-1}=x,x_{i}=x^{\prime }\}|$, and for Model 3, if $q=(x,y,x^{\prime })$, then $♯q=|\{i\in \bar{2,m}|x_{i-1}=x,y_{i-1}=y,x_{i}=x^{\prime }\}|$ . Similarly, we define ♯(q,a) and $♯(q,a,q^{\prime })$ for a∈Σ and $q,q^{\prime }\in Q$ as follows:

**Model 1**: $$q=x,a=y,q^{\prime }=x^{\prime },$$

$$♯\left(q,a\right)=|\left\{i\in \bar{1,m}|x_{i}=x,y_{i}=y\right\}|,$$

$$♯\left(q,a,q^{\prime }\right)=\left|\left\{i\in \bar{1,m-1}|x_{i}=x,y_{i}=y,x_{i+1}=x^{\prime }\right\}\right|.$$

**Model 2**: $$q=\left(x^{\prime \prime },x\right),a=y,q^{\prime }=\left(x,x^{\prime }\right),$$

$$♯\left(q,a\right)=|\left\{i\in \bar{2,m}|x_{i-1}=x^{\prime \prime },x_{i}=x,y_{i}=y\right\}|,$$

$$♯\left(q,a,q^{\prime }\right)=\left|\left\{i\in \bar{2,m-1}|x_{i-1}=x^{\prime \prime },x_{i}=x,x_{i+1}=x^{\prime },y_{i}=y\right\}\right|.$$

**Model 3**: $$q=\left(x^{\prime \prime },y^{\prime \prime },x\right),a=y,q^{\prime }=\left(x,y,x^{\prime }\right),$$

$$♯\left(q,a\right)=|\left\{i\in \bar{2,m}|x_{i-1}=x^{\prime \prime },x_{i}=x,y_{i-1}=y^{\prime \prime },y_{i}=y\right\}|,$$

$$♯\left(q,a,q^{\prime }\right)=\left|\left\{i\in \bar{2,m-1}|x_{i-1}=x^{\prime \prime },x_{i}=x,x_{i+1}=x^{\prime },y_{i-1}=y^{\prime \prime },y_{i}=y\right\}\right|.$$

Note that, in the case of Models 2 and 3, $♯(q,a,q^{\prime })$ is zero by definition if the last element of state *q* is different than the first element of state $q^{\prime }$. Next, we estimate the values of *ι* and ϕ with the following formulas (we use Laplace smoothing to avoid zero values in the testing phase for elements that never appeared in training):

$$\iota \left(q\right):=\frac{♯q+1}{m+|Q|},\;\;\;\;\;\;\phi \left(q,a,q^{\prime }\right):=\frac{♯(q,a,q^{\prime })+1}{♯q+|Q|\times |\Sigma |}$$

It is easy to see that P(a∣q), the probability of performing the action *a* when in state *q*, defined as $\sum_{q^{\prime }\in Q}\phi (q,a,q^{\prime })$, becomes (♯(q,a)+|Q|)/(♯q+|Q|×|Σ|) (see [24] for a survey on learnings PFAs).

### 2.4. Evaluation Metrics

In the case of BR, the goal is to identify which was the strategy employed by an agent *A* using the learning trace $T=[\left(x_{1},y_{1}\right),\ldots ,\left(x_{m},y_{m}\right)]$ that the agent produced. To this end, we train a PFA $A_{k}$ for each available planned strategy, we compute the value $P_{A_{k}}\left(\alpha ,\pi^{M}\right)$ and return $\arg\;\max_{k}\left\{P_{A_{k}}\left(\alpha ,\pi^{M}\right)\right\}$, where $\alpha =(y_{1},\ldots ,y_{m})$ and $\pi^{M}=\left(q_{1}^{M},\ldots ,q_{m}^{M}\right)$. The value of $q_{i}^{M}$ depends on the amount *M* of memory used: $q_{i}^{1}=x_{i}$ for Model 1, $q_{i}^{2}=\left(x_{i-1},x_{i}\right)$ for Model 2 and $q_{i}^{3}=\left(x_{i-1},y_{i-1},x_{i}\right)$ for Model 3.

In practice, we use the values $P_{A_{k}}\left(\alpha ,\pi^{M}\right)$ to measure the distance between the behavior *B* exhibited by agent *A* (through the learning trace *T*) and the behavior $B^{\prime }$ of the strategy modeled by automaton $A_{k}$ by computing the negative log-probability:

(5) $$R_{A_{k}}^{M}\left(T\right)=-\frac{1}{m}\log P_{A_{k}}\left(\alpha ,\pi^{M}\right)$$

This value can be interpreted as a Monte Carlo approximation of the crossed entropy between behaviors *B* and $B^{\prime }$, known in the literature as Vapnik’s risk (see [19]). Obviously, maximizing $P_{A_{k}}\left(\alpha ,\pi^{M}\right)$ is the same as minimizing $R_{A_{k}}^{M}\left(T\right)$ and for practical reasons (in order to avoid underflow and because adding is faster than multiplying), we use distances instead of probabilities.

In the case of BC, we are interested in assessing the quality of the models proposed (that is, we would like to know how well does the cloned agent behave on previously unseen data). For this purpose, we use two different metrics, which we detail below.

Predictive Accuracy. This is a standard measure for classification tasks. Let M be the model trained by one of the learning algorithms using the trace obtained by observing an agent *A* (that follows a certain strategy) on a fixed set of maps. This model can be either deterministic (in which case, there is only one possible action at any point in time) or stochastic (the action is chosen randomly according to some probability distribution).

Now, let $T=[\left(x_{1},y_{1}\right),\ldots ,\left(x_{m},y_{m}\right)]$ be the trace of the agent *A* on a different previously unseen map. The predictive accuracy $Acc_{M}\left(T\right)$ is measured as follows:

(6) $$\begin{array}{c} Acc_{M}\left(T\right)=\frac{1}{m}\sum_{i=1}^{m}♯(M,T,i),\;♯\left(M,T,i\right)=\left\{\begin{array}{cc} 1, & \text{if}\;\;\;M\left(\left[x_{i-1},y_{i-1},\right]x_{i}\right)=y_{i}\\ 0, & \text{otherwise} \end{array}\right. \end{array}$$

where $M(\left[x_{i-1},y_{i-1},\right]x_{i})$ represents the action predicted by the model M for the state $x_{i}$, possibly knowing previous state and action. If M is a stochastic model, $M(\left[x_{i-1},y_{i-1},\right]x_{i})$ is a random variable over Y with the probability distribution ${\left\{P\left(a|q\right)\right\}}_{a\in Y}$, where $q=x_{i}$ for Model 1, $q=(x_{i-1},x_{i})$ for Model 2 and $q=(x_{i-1},y_{i-1},x_{i})$ for Model 3.

Monte Carlo Distance. To assess the adequacy of a model M in reproducing the behavior of an agent *A*, we propose a Monte Carlo-like measure based on estimating the crossed entropy between the probability distributions associated with both the model M and the agent *A*. More concretely, let $T=[\left(x_{1},y_{1}\right),\ldots ,\left(x_{n},y_{n}\right)]$ be a trace generated (the model predicts the next action, but the next state is given by the actual configuration of the map; in the case that it is impossible to perform a certain action because of an obstacle, the agent does not change its location) according to model M on a fixed map (different than the one used in training), and let $T^{\prime }=\left[\left(x_{1}^{\prime },y_{1}^{\prime }\right),\ldots ,\left(x_{m}^{\prime },y_{m}^{\prime }\right)\right]$ be the trace generated by the agent on the same map. We define the Monte Carlo distance between model M and agent *A* as follows (estimated through traces *T* and $T^{\prime }$):

(7) $$H\left(T,T^{\prime }\right)=-\frac{1}{n}\sum_{i=1}^{n}log\left[\frac{1}{m}\sum_{j=1}^{m}I_{\left\{o_{j}^{\prime }\right\}}\left(o_{i}\right)\right]$$

where $o_{i}=\left(\left[x_{i-1},y_{i-1},\right]x_{i},y_{i}\right)$ and $o_{i}^{\prime }=\left(\left[x_{i-1}^{\prime },y_{i-1}^{\prime },\right]x_{i}^{\prime },y_{i}^{\prime }\right)$ (depending on the model used, we may store into our observations information about previous state and action). Here, $I_{\left\{o_{j}^{\prime }\right\}}$ means the indicator function of set $\left\{o_{j}^{\prime }\right\}$. The previous measure in Equation (7) is obviously empirical. For large enough traces, it approximates the true cross entropy between the behavior corresponding to model M and the behavior exhibited by agent *A*. Using Laplace smoothing, the previous formula becomes:

(8) $$H\left(T,T^{\prime }\right)=-\frac{1}{n}\sum_{i=1}^{n}log\left[\frac{\sum_{j=1}^{m}I_{\left\{o_{j}^{\prime }\right\}}\left(o_{i}\right)+1}{m+|Q|\times |Y|}\right]$$

## 3. Experiments

We have run our experiments with a simulator of a simplified version of a Roomba (iRobot, Bedford, MA, USA), which is a series of autonomous robotic vacuum cleaners sold by iRobot (According to the company’s website (http://www.irobot.com), iRobot Corporation is an American advanced technology company founded in 1990 by Massachusetts Institute of Technology roboticists. More than 14 million home robots have been sold worldwide. Roomba was introduced in 2002). The original Roomba vacuum cleaner uses a set of basic sensors in order to perform its tasks. For instance, it is able to change direction whenever it encounters an obstacle. It uses two independently operating wheels that allow 360 degree turns in place. Additionally, it can adapt to perform other more creative tasks using an embedded computer in conjunction with the Roomba Open Interface.

### 3.1. Training Maps

**Figure 1:**
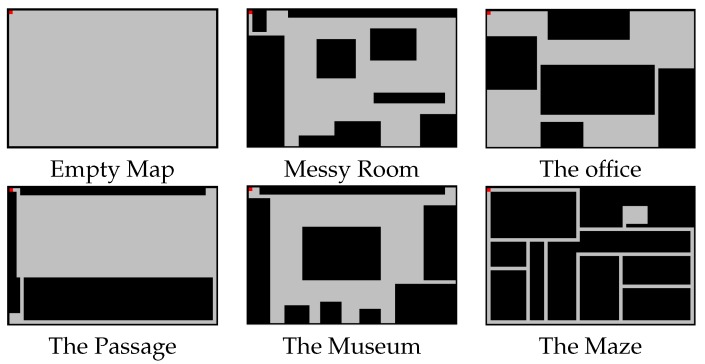
Training Maps, where obstacles are shown in black. The starting position of the vacuum cleaner is shown in red.

The environment in which the agent moves is a 40 × 60 rectangle surrounded by walls, which may contain all sorts of obstacles. For testing, we have randomly generated obstacles on an empty map. Below, we briefly explain the six maps used in the training phase (they are visually represented in Figure 1). Each of them is meant to represent a real-life situation, as indicated by their title.

- **Empty Map.** The empty map consists of a big empty room with no obstacles.
- **Messy Room.** The messy room simulates an untidy teenager bedroom, with all sorts of obstacles on the floor, and with a narrow entry corridor that makes the access to the room even more challenging for any “hostile intruder”.
- **The Office.** The office map simulates a space in which several rooms are connected to each other by small passages. In this case, obstacles are representing big furniture such as office desks or storage cabinets.
- **The Passage.** The highlight of this map is an intricate pathway that leads to a small room. The main room is big and does not have any obstacle in it.
- **The Museum.** This map is intended to simulate a room from a museum, with the main sculpture in the center, and with several other sculptures on the four sides of the room, separated by small spaces.
- **The Maze.** In this map, there are many narrow pathways with the same width as the agent. It also contains a little room, which is difficult to find.

### 3.2. Agent Strategies

In our experiments, the simulation time is discrete, and, at each time step, the agent can take one out of these four actions: up, down, left and right, with their intuitive effect. The agent perceives the world through the input variable *X* having four different binary components (up, down, left, right), each one of them identifying what the vacuum cleaner can see in each direction (obstacle, no obstacle). We have designed a series of strategies with different complexities. When describing a strategy, we must define the behavior of the agent in a certain situation (its state $X_{t}$) that depends on the configuration of obstacles in its vicinity (prefix Rnd is used for stochastic strategies).

- Walk. The agent always performs the same action in a given state. As an example, a possible strategy could be to go Right whenever there are no obstacles, and Up whenever there is only one obstacle to the right (stationary deterministic behavior of Type 2, it only depends on current state $X_{t}$).
- Rnd_Walk. In this strategy, the next move is selected randomly from the set of available moves. For example, an agent that has obstacles to the right and to the left can only move Up or Down, but there is no predefined choice between those two (stationary stochastic behavior of Type 2, it only depends on current state $X_{t}$).
- Crash. In this strategy, the agent should perform the same action as in the previous time step (if possible). Whenever it encounters a new obstacle in its way, the agent must choose a certain predefined action. Therefore, it needs to have information about its previous action in order to know where to move (stationary deterministic behavior of Type 3, it depends on current state $X_{t}$ and previous action $Y_{t-1}$).
- Rnd_Crash. This strategy allows the agent to take a random direction when it crashes with an obstacle. The main difference with the Rnd_Walk is that in Rnd_Crash the agent does not change direction if it does not encounter an obstacle in its way (stationary stochastic behavior of Type 3, it depends on current state $X_{t}$ and previous action $Y_{t-1}$).
- ZigZag. It consists of different vertical movements in two possible directions, avoiding the obstacles. It has an internal state that tells the robot if it should advance towards the left or the right side with this vertical movements: it initially goes towards the right side, and once it reaches one of the right corners, the internal state changes so that the robot will start moving toward the left side (stationary deterministic behavior of Type 3, it depends on current state $X_{t}$, previous action $Y_{t-1}$ and internal state $C_{t}$).
- Rnd_ZigZag. This strategy is similar to the previous one, with the only difference that, once it reaches a corner, the internal state could either change its value or not, and this is randomly assigned (stationary stochastic behavior of Type 3, it depends on current state $X_{t}$, previous action $Y_{t-1}$ and internal state $C_{t}$).

### 3.3. Trace and Performance Evaluation

We work with an agent represented by a simplified version of a Roomba robot. In our implementation, although it is possible for the agent to start anywhere, the traces we generate are always with the agent starting in the top-left corner of the map. We use the strategies explained in Section 3.2 and the maps described in Section 3.1. For each of the six strategies, we have generated six traces of 1500 time steps (one for each map) and merged them together into one single trace, which was used as training data.

### 3.4. Behavioral Recognition Experimentation

In order to determine whether our approach leads to a correct identification of the agent’s strategy, we performed the following experiment. First, we generated 100 random maps (each of them having a total of 150 obstacles). Then, we generated a family of traces (each of them of 1500 time steps) for each pair strategy/map: ${\left(T_{n}^{i}\right)}_{i\in \{1,\ldots ,100\},n\in \{1,\ldots ,6\}}$ and we computed the log-normalized distance $R_{A_{m}}^{M}\left(T_{n}^{i}\right)$ between the observation trace $T_{n}^{i}$ and the automaton $A_{m}$ (see Equation (5)). The average value $R_{m,n}^{M}=\sum_{i=1}^{100}R_{A_{m}}^{M}\left(T_{n}^{i}\right)/100$ of these distances is reported in Table 2, in the (m,n)-th cell.

Our system classifies the testing task represented by column *n* as being generated by the automaton $A_{k}$ such that $k=\arg\;\min_{m}R_{m,n}^{M}$ (minimizing distance maximizes trace probability). The smallest value of each column is marked in bold.

Analysis of the results indicates that the PFA recognition system is able to correctly identify the three random strategies (Rnd_Walk, Rnd_Crash and Rnd_ZigZag). However, the system most often fails when recognizing the respective underlying deterministic strategies (Walk, Crash and ZigZag). In addition, note that the deterministic versions of the random behaviors Crash and ZigZag are not confused with each other but each of them is most of the times classified by the system as its corresponding non-deterministic version (Crash is classified as Rnd_Crash and ZigZag as Rnd_ZigZag for all three models). Moreover, Walk is, on average, correctly classified by Models 2 and 3.

In Table 3, we present the confusion matrix. This is a specific table layout that allows the visualization of the performance of a supervised learning algorithm (see [16]). The numerical value $C_{m,n}^{M}$ placed in the (m,n)-th cell of this matrix is the empirical probability of the *n*-th task to be classified as the *m*-th task, that is, the percentage of the learning traces produced using strategy *n* that are recognized as being produced by strategy *m*. More precisely,

$$C_{m,n}^{M}=\frac{\left|\{i\in \left\{1,\ldots ,n_{tests}\right\}|m=\arg\;\min_{k}R_{A_{k}}^{M}\left({\bar{T}}_{n}^{i}\right)\}\right|}{n_{tests}}$$

The diagonal of this matrix reflects the empirical probabilities of right classification and the sum of the other rows different from the diagonal element is the probability of error.

We observe that this second table confirms the conclusions of the first one, with one notable exception: Models 2 and 3 seem to be more prone to confuse the Walk strategy with Rnd_Crash, while Model 1 is the one with highest rates of success. Note that the percentage of right classification is not negligible for deterministic strategies of Type 3 (around 0.20 in the case of Crash for all three models, and between 0.16 and 0.39 for ZigZag).

### 3.5. Behavioral Cloning Experimentation

As in the case of BR, we used a single trace containing 9000 time steps for each strategy to train our models. For testing, we used the same set of 100 randomly generated maps.

#### 3.5.1. Predictive Accuracy

The numbers in the three tables of Table 4 represent average values of the predictive accuracy (see Equation (6)) computed for each of the randomly generated maps. Note that the only stochastic model is the PFA.

Analyzing the results, we can see that our hierarchy of models behaves as expected: Type 3 behavior is very well captured by Model 3 (the one that uses information about both previous state and previous action), while Type 2 deterministic behavior is better explained by Model 1 (in which we only take into account the current state). Note that, even though, intuitively, the more info we have the best we can predict, in the case of Type 2 behavior, using this extra information can do more harm than good. Another anticipated result that was experimentally confirmed is that Model 1 would be very good in predicting the Walk strategy because the agent always performs the same action in a given state. A surprising conclusion that can be drawn is that, while Rnd_Walk should be the most unpredictable strategy of all, in the case of Models 2 and 3, most of our classifiers have even worse accuracy for the Walk strategy. It is worth noting the high accuracy rates of Model 3 for Rnd_Crash, Rnd_ZigZag and ZigZag, all Type 3 strategies. Furthermore, this model gives somewhat lower but still satisfactory prediction rates for the Crash strategy.

A PFA is the best option when predicting the behavior of Walk and Zigzag strategies (both in their deterministic and stochastic versions) using a minimal amount of memory. In addition, although, intuitively, a stochastic model should be better than a deterministic one in describing the behavior of a random process. According to the predictive accuracy metric, the advantage of using PFAs apparently has to do more with the amount of memory used and not with the nature of the underlying process.

#### 3.5.2. Monte Carlo Distance

Predictive accuracy, however, is not a very good metric for BC when behaviors are non-deterministic. Consider, for example, the extreme case of cloning a random agent. Using the predictive accuracy metric, the highest accuracy a learning agent could expect is 0.25 (if there are four possible actions), even when the behavior is perfectly cloned. Thus, the Monte Carlo distance metric is a more adequate metric when comparing stochastic behaviors.

We used the same set of 100 randomly generated maps. For each of the classifiers (DT, PNN, KNN, RProp, NB) and, for each of the six predefined strategies (Walk, Rnd_Walk, Crash, Rnd_Crash, ZigZag, Rnd_ZigZag), we generated the trace *T* produced by an agent whose next action is dictated by the classifier on each of the randomly generated maps (each trace would contain at most 1500 observations). Note that, since the strategy is a learned one, it could happen that the action suggested by the model is not feasible (for example, the model says to go up even if there is an obstacle in that direction). Therefore, one may end up having empty traces (marked by a dash in Table 5). On the other hand, we generated the trace $T^{\prime }$ of each of the six predefined strategies on those maps (in this case, all traces have 1500 observations). In Table 5, we present the average of $H(T,T^{\prime })$ for each pair classifier/strategy (see Equation (8)). For stochastic models, the next action, instead of being predefined, is obtained by sampling according to the probability distribution given by the trained model.

According to this metric, PFAs are globally the best tool when less information is used (Models 1 and 2), being outperformed by DTs in the case in which the model of the learner also takes into consideration the previous action (Model 3). Note that PFAs are mostly better than other classifiers when learning random strategies. One surprising conclusion that can be drawn is that NB seem to be the best tool for the Walk strategy when the learner is allowed to use knowledge about the past (Models 2 and 3).

Finally, for each model, each strategy and each machine learning tool, we report the number of times the given tool was the best one. Since we have 100 different maps, this number is always between 0 and 100. In the case of equal Monte Carlo distance score, we assigned a corresponding fraction to each of the ‘winning’ models. For example, if there are three tools with the same score, each of them receives 1/3 points for that particular map (therefore, we end up having real numbers other than integers).

Table 6 confirms PFAs as being the best tool in the case of Model 1, this time even for the Crash strategy. In the case of Model 2, it is the best overall tool and the most reliable one for random strategies. In addition, note that the frequency with which it returns the best score is very close to the NB one for Walk and to the DT one for Zigzag. Again, it is the the third model where its use does not pay off, being outperformed by DTs when the strategy to be cloned is of Type 3.

## 4. Conclusions

### 4.1. Discussion

Three of the major open challenges in Learning from Observation are (1) devising training regimes that address the particularities that make LfO different from supervised learning (namely that LfO violated the i.i.d. assumption) [25]; (2) devising approaches to handle long-range variable interactions (actions depending on past states); and (3) devising performance metrics that adequately characterize the performance of LfO agents. The work presented in this paper (in addition to being a general probabilistic framework that can handle both Behavior Recognition and Behavioral Cloning) constitutes a contribution to several of those challenges.

Specifically, we presented an empirical study showing the learning performance of PFAs under different assumptions over the amount of past memory used for learning, showing that considering past states can significantly improve learning performance when long-range dependencies exist. However, when learning behaviors where such long-range dependencies do not exist, considering past states actually hinders performance. Additionally, building on our previous work [19], we showed how traditional supervised performance metrics, such as classification accuracy, are not enough to capture the performance of LfO agents, and proposed a metric based on Vapnik’s risk.

Concerning experimental results for Behavior Recognition, we have shown that our approach using PFAs correctly identifies tasks performed by the agent whenever those tasks have a certain random component. The inference technique is based on the greatest likelihood probability value generated by the PFAs of the model. One of the strengths of our approach is that it captures the stochastic aspect of behaviors. On the other hand, we observed that this technique has difficulties in distinguishing between a certain strategy and a similar strategy perturbed with some degree of randomness.

Concerning Behavioral Cloning, we compared our approach against supervised learning classifiers that predict the action of the agent in a given state (deterministic models). Then, we trained a PFA to estimate the probabilities of the agent’s actions in a given state (a stochastic model). In both cases, we measured the predictive accuracy on new unseen data: supervised learning approaches seemed to perform better according to classification accuracy. However, when comparing the trace of the learned strategy with the trace of the original agent on a randomly generated map using a Monte Carlo distance metric, we could experimentally check that PFAs had actually learned a better model of the agent’s behavior in general. This highlights both the need to use appropriate models to learn certain behaviors (e.g., stochastic models for stochastic behaviors), and the use of appropriate performance metrics for the evaluation of LfO algorithms.

### 4.2. Future Work

A major challenge in both behavior recognition and behavioral cloning approaches, such as the ones explored in this paper, is determining the amount of ‘memory’ that the learning agent should have access to in order to model the behavior at hand. In our experimental results, we have seen that, for behaviors that only require considering the current state, using Model 1 (which only considers the current state) results in better learning performance. More complex models (like Models 2 and 3 considered above) allow learning more complex behaviors, but they should be used only if necessary. Devising strategies for determining the amount of past memory required to learn a task remains an open problem, which we plan to address in our future work.

Concerning scalability, the major computational limitation of the proposed learning machines is the amount of memory required by the trained automaton. A possible solution we plan to investigate in our future work is to employ only a small number of non-observable internal states. Another possible approach is the use of some notion of context learning [26] in order to reduce the number of possible states.

We also plan to extend this methodology to handle continuous state and action spaces. This would allow modeling more realistic Type of robots, which can perform rotations of different angles and whose position is represented by real values on the map. This would allow us to consider behaviors such as the outward-moving spiral of the Roomba robot. Future work also contemplates the usage of probabilistic transducers to take into account the input-output (state-action) nature of the observations composing the learning traces in the observation scenario.

## Figures and Tables

**Table 1:** Training examples for LfO.

Model 1		Model 2			Model 3			
1st	Class	1st	2nd	Class	1st	2nd	3rd	Class
$x_{1}$	$y_{1}$	−	$x_{1}$	$y_{1}$	−	−	$x_{1}$	$y_{1}$
$x_{2}$	$y_{2}$	$x_{1}$	$x_{2}$	$y_{2}$	$x_{1}$	$y_{1}$	$x_{2}$	$y_{2}$
…	…	…	…	…	…	…	…	…
$x_{m}$	$y_{m}$	$x_{m-1}$	$x_{m}$	$y_{m}$	$x_{m-1}$	$y_{m-1}$	$x_{m}$	$y_{m}$

**Table 2:** Distance matrix.

$R_{m,n}^{1}$ values (Model 1)						
9000/1500 obs	Walk	Rnd_Walk	Crash	Rnd_Crash	ZigZag	Rnd_ZigZag
Walk	8.4267	9.2504	7.3178	7.8728	8.9871	9.0486
Rnd_Walk	8.9414	**3.9751**	4.3265	3.8846	4.6463	4.6070
Crash	8.4996	6.5079	4.7726	5.8376	4.3072	4.3743
Rnd_Crash	**8.4245**	4.1331	**3.9854**	**3.4244**	4.3935	4.3924
Zigzag	9.1459	7.1087	5.3849	5.8159	3.9715	3.9815
Rnd_Zigzag	9.1683	7.1035	5.4043	5.7853	**3.9563**	**3.9591**
$R_{m,n}^{2}$ **values (Model 2)**						
**9000/1500 obs**	**Walk**	**Rnd_Walk**	**Crash**	**Rnd_Crash**	**ZigZag**	**Rnd_ZigZag**
Walk	**9.7283**	9.8276	8.5274	8.7760	9.7278	9.7654
Rnd_Walk	9.8016	**4.9923**	6.4832	5.8042	6.0120	6.0783
Crash	9.8502	8.0886	6.8461	7.4848	6.4688	6.5510
Rnd_Crash	9.8487	5.6960	**6.1228**	**5.2204**	6.3857	6.4331
Zigzag	9.8663	8.1203	7.8902	8.4213	5.5873	5.6445
Rnd_Zigzag	9.8681	8.1002	7.8278	8.3599	**5.5506**	**5.5981**
$R_{m,n}^{3}$ **values (Model 3)**						
**9000/1500 obs**	**Walk**	**Rnd_Walk**	**Crash**	**Rnd_Crash**	**ZigZag**	**Rnd_ZigZag**
Walk	**11.7101**	11.8912	10.5335	10.7006	11.8687	11.8792
Rnd_Walk	11.8415	**7.1282**	8.8885	8.2754	8.2794	8.3277
Crash	11.8359	11.4252	8.4840	9.0659	8.1677	8.2454
Rnd_Crash	11.8261	10.7019	**7.5571**	**6.5445**	7.8168	7.8871
Zigzag	11.8808	11.2667	9.2245	9.6458	6.7169	6.8174
Rnd_Zigzag	11.8824	11.2563	9.1614	9.5850	**6.6728**	**6.7602**

**Table 3:** Confusion matrix.

$C_{m,n}^{1}$ values (Model 1)						
9000/1500 obs	Walk	Rnd_Walk	Crash	Rnd_Crash	ZigZag	Rnd_ZigZag
Walk	**0.71**	0.00	0.00	0.00	0.00	0.00
Rnd_Walk	0.12	**0.99**	0.10	0.00	0.00	0.01
Crash	0.03	0.00	0.20	0.00	0.03	0.04
Rnd_Crash	0.01	0.01	**0.63**	**0.99**	0.10	0.09
Zigzag	0.13	0.00	0.03	0.01	0.39	0.24
Rnd_Zigzag	0.00	0.00	0.04	0.00	**0.48**	**0.62**
**$C_{m,n}^{2}$ values (Model 2)**						
9000/1500 obs	Walk	Rnd_Walk	Crash	Rnd_Crash	ZigZag	Rnd_ZigZag
Walk	0.28	0.00	0.04	0.01	0.01	0.01
Rnd_Walk	0.05	**1.00**	0.24	0.01	0.00	0.00
Crash	0.24	0.00	0.21	0.01	0.03	0.03
Rnd_Crash	**0.42**	0.00	**0.43**	**0.97**	0.03	0.01
Zigzag	0.01	0.00	0.01	0.00	0.18	0.05
Rnd_Zigzag	0.00	0.00	0.07	0.00	**0.75**	**0.90**
**$C_{m,n}^{3}$ values (Model 3)**						
9000/1500 obs	Walk	Rnd_Walk	Crash	Rnd_Crash	ZigZag	Rnd_ZigZag
Walk	0.27	0.00	0.03	0.01	0.01	0.01
Rnd_Walk	0.05	**1.00**	0.01	0.00	0.00	0.00
Crash	0.07	0.00	0.20	0.01	0.03	0.02
Rnd_Crash	**0.60**	0.00	**0.64**	**0.98**	0.01	0.01
Zigzag	0.01	0.00	0.02	0.00	0.16	0.03
Rnd_Zigzag	0.00	0.00	0.10	0.00	**0.79**	**0.93**

**Table 4:** Predictive accuracy for the generated maps (higher is better).

Model 1	PFA	DT	PNN	KNN	RProp	NB
Walk	**0.977**	0.925	0.924	0.925	0.452	0.504
Rnd_Walk	**0.276**	**0.276**	0.275	**0.276**	0.270	0.253
Crash	0.571	**0.629**	**0.629**	0.626	0.561	0.336
Rnd_Crash	0.388	**0.398**	0.392	0.394	0.381	0.381
Zigzag	**0.485**	0.478	0.478	0.475	0.454	0.456
Rnd_Zigzag	**0.485**	0.467	0.468	0.464	0.442	0.447
Average	**0.530**	0.529	0.528	0.527	0.427	0.396
**Model 2**	**PFA**	**DT**	**PNN**	**KNN**	**RProp**	**NB**
Walk	0.288	**0.516**	0.079	0.124	0.448	0.078
Rnd_Walk	0.270	**0.276**	0.269	0.272	0.267	0.267
Crash	0.509	**0.612**	0.570	0.578	0.547	0.328
Rnd_Crash	0.391	0.419	0.411	**0.421**	0.392	0.370
Zigzag	0.522	0.554	**0.556**	0.543	0.504	0.460
Rnd_Zigzag	0.528	**0.584**	0.558	0.574	0.513	0.451
Average	0.418	**0.493**	0.407	0.419	0.445	0.326
**Model 3**	**PFA**	**DT**	**PNN**	**KNN**	**RProp**	**NB**
Walk	**0.288**	0.079	0.080	0.119	0.080	0.071
Rnd_Walk	0.267	0.276	0.267	0.271	**0.278**	0.271
Crash	0.702	**0.815**	0.705	0.725	0.758	0.563
Rnd_Crash	0.923	**0.945**	0.909	0.929	0.930	0.929
Zigzag	0.881	**0.960**	0.885	0.910	0.871	0.836
Rnd_Zigzag	0.880	**0.961**	0.882	0.930	0.876	0.815
Average	0.657	**0.673**	0.621	0.647	0.632	0.581

**Table 5:** Monte Carlo distance between original and cloned behavior (lower is better).

Model 1	PFA	DT	PNN	KNN	RProp	NB
Walk	**0.854**	0.866	0.962	0.866	4.836	4.797
Rnd_Walk	**2.487**	5.130	5.062	5.062	-	-
Crash	3.012	2.599	2.599	**2.574**	3.119	5.572
Rnd_Crash	**3.067**	3.853	3.762	3.823	3.343	3.767
Zigzag	**2.261**	3.497	3.608	3.497	3.316	4.324
Rnd_Zigzag	**2.357**	3.506	3.591	3.506	4.354	4.354
Average	**2.340**	3.242	3.264	3.221	3.794	4.563
**Model 2**	**PFA**	**DT**	**PNN**	**KNN**	**RProp**	**NB**
Walk	6.165	7.072	7.274	6.718	6.159	**6.033**
Rnd_Walk	**5.385**	6.886	6.958	7.189	7.538	7.575
Crash	5.538	6.460	5.408	5.760	**4.817**	-
Rnd_Crash	**4.750**	5.157	5.602	5.757	4.912	-
Zigzag	4.818	**4.191**	4.501	4.336	-	7.663
Rnd_Zigzag	**4.759**	4.502	4.727	4.544	4.524	7.683
Average	**5.236**	5.711	5.745	5.718	5.590	7.239
**Model 3**	**PFA**	**DT**	**PNN**	**KNN**	**RProp**	**NB**
Walk	6.961	8.082	7.953	7.524	6.955	**6.830**
Rnd_Walk	**6.940**	7.894	7.941	7.983	7.978	8.348
Crash	6.000	**4.797**	6.924	6.685	5.613	5.613
Rnd_Crash	5.470	**4.986**	6.324	5.325	5.708	-
Zigzag	5.914	**4.561**	5.324	4.903	5.871	8.460
Rnd_Zigzag	5.845	**4.220**	5.416	4.934	5.234	8.459
Average	6.188	**5.757**	6.647	6.226	6.227	7.542

**Table 6:** Empirical distribution of the winning tool (smallest Monte Carlo distance).

Model 1	PFA	DT	PNN	KNN	RProp	NB
Walk	**31.50**	22.50	21.50	22.50	1.00	1.00
Rnd_Walk	**99.00**	1.00	0.00	0.00	0.00	0.00
Crash	**43.30**	10.30	10.30	10.30	24.30	1.50
Rnd_Crash	**63.33**	3.00	5.00	4.33	21.00	3.33
Zigzag	**95.17**	0.92	0.92	0.92	0.92	1.17
Rnd_Zigzag	**93.17**	1.50	1.50	1.50	1.17	1.17
Average	**70.91**	6.54	6.54	6.59	8.06	1.36
**Model 2**	**PFA**	**DT**	**PNN**	**KNN**	**RProp**	**NB**
Walk	29.95	21.25	4.70	5.12	7.95	**31.03**
Rnd_Walk	**58.67**	16.50	16.00	4.50	2.17	2.17
Crash	9.50	22.00	22.33	8.83	**37.17**	0.17
Rnd_Crash	**31.00**	24.00	7.50	4.50	33.00	0.00
Zigzag	26.50	**30.50**	17.33	22.33	1.67	1.67
Rnd_Zigzag	**34.33**	15.92	8.92	17.08	22.08	1.67
Average	**31.66**	21.69	12.80	10.39	17.34	6.12
**Model 3**	**PFA**	**DT**	**PNN**	**KNN**	**RProp**	**NB**
Walk	**31.12**	16.83	8.28	5.45	8.37	29.95
Rnd_Walk	**49.00**	15.50	10.00	5.50	15.00	5.00
Crash	13.00	**50.00**	12.33	10.33	7.17	7.17
Rnd_Crash	11.67	**37.17**	10.67	20.67	19.83	0.00
Zigzag	7.42	**39.67**	11.42	28.42	11.42	1.67
Rnd_Zigzag	8.67	**47.67**	9.17	22.17	10.67	1.67
Average	20.15	**34.47**	10.31	15.42	12.08	7.58

## Abbreviations

The following abbreviations are used in this manuscript:

PFA: Probabilistic Finite Automaton
SAF: Semi-Automated Forces
CGF: Computer-Generated Forces
RL: Reinforcement Learning
LfO: Learning from Observation
ALVINN: Autonomous Land Vehicle In a Neural Network
SOAR: State, Operator And Result
DNA: DeoxyriboNucleic Acid
AR: Activity Recognition
HAR: Human Activity Recognition
BR: Behavioral Recognition
BC: Behavioral Cloning
DT: Decision Tree
kNN: K-Nearest Neighbor
NB: Naive Bayes
PNN: Probabilistic Neural Network
MICINN: Ministerio de Ciencia e Innovación
NSF: National Science Foundation
